# Determinants of adverse maternal and perinatal outcomes in severe preeclampsia and eclampsia in a low-resource setting, Mpilo Central Hospital, Bulawayo, Zimbabwe

**DOI:** 10.1186/s13104-019-4334-9

**Published:** 2019-05-28

**Authors:** Solwayo Ngwenya, Brian Jones, Desmond Mwembe

**Affiliations:** 1 Department of Obstetrics & Gynaecology, Mpilo Central Hospital, P.O. Box 2096, Vera Road, Mzilikazi, Bulawayo, Matabeleland Zimbabwe; 2 Royal Women’s Clinic, 52A Cecil Avenue, Hillside, Bulawayo, Zimbabwe; 3 National University of Science and Technology, Medical School, P. O. Box AC 939, Ascot, Bulawayo, Matabeleland Zimbabwe

**Keywords:** Severe preeclampsia, Eclampsia, Determinants, Adverse outcomes, Low-resource setting, Mpilo Central Hospital

## Abstract

**Objective:**

Severe preeclampsia and eclampsia have dire consequences for both maternal and neonatal health. The objective of this study was to identify determinants of adverse maternal and perinatal outcomes in severe preeclampsia and eclampsia.

**Results:**

Binary logistic regression showed the following were significantly associated with adverse maternal outcomes; mothers who had a baby born at 27–29^+6^ weeks of gestation were 8 times more likely to be associated with adverse maternal outcomes compared to mothers who gave birth at 37–39^+6^ weeks’ of gestation (OR 8.187, 95% CI 1.680–39.911, *p *= 0.02), holding other variables constant. Platelet count was also statistically significant for adverse maternal outcome. Mothers with platelet counts of 0–49 × 10^9^/l were 46 times more likely to be associated with adverse maternal outcome compared to mothers with normal counts of more than 150 × 10^9^/l (OR 46.429, 95% CI 17.778–121.253, *p *= 0.001). The following determinants were significantly associated with adverse perinatal outcomes. Mothers with platelet counts of 0–49 × 10^9^/l were 4 times more likely to be associated with adverse perinatal outcomes compared to mothers with platelet counts of above 150 × 10^9^/l (OR 3.690, 95% CI 1.752–7.775, *p *= 0.001).

## Introduction

According to some German authors, the first reports referring to eclampsia date from 2200 BC, observed in papyri of ancient Egypt [1]. The incidence of preeclampsia remains underestimated due to underreporting [2].

Severe preeclampsia is defined once there is high blood pressure (i.e. BP > 160–170/100–110), heavy proteinuria of > 3–5 g/24 h, and/or the occurrence of symptoms, such as headache or visual disturbances [3]. Eclampsia occurs when a pregnant woman with features of severe preeclampsia has a grand mal seizure without prior history of epilepsy.

Severe preeclampsia and eclampsia have dire consequences for both maternal and neonatal health, with 50,000–100,000 annual maternal deaths attributable to these conditions globally, as well as 500,000 fetal and neonatal deaths [4].

Globally, the three most common causes of maternal deaths are haemorrhage, hypertensive disorders and sepsis, accounting for more than half of maternal deaths worldwide as of 2010 [5]. Developing countries accounted for approximately 99% (302,000) of the global maternal deaths in 2015, with Sub-Saharan Africa alone accounting for roughly 66% (201,000) [6].

In Zimbabwe, hypertensive disorders were the third leading cause of maternal deaths as of the last 2007 maternal demographic study [7]. In Brazil the overall incidence of preeclampsia was 1.5% [8]. There are reports that HIV-infected women are at increased risk of preeclampsia [9].

## Main text

### Methods

#### Study type, setting and participants

This study was done at Mpilo Central Hospital, a tertiary government unit. It was a retrospective cross-sectional study, from January 1, 2016 to December 31, 2018, some of the participants overlapped with a study done on severe preeclampsia and eclampsia published in 2017 [10]. Patients included in the study were those with severe preeclampsia and either were symptomatic of severe preeclampsia or deranged biochemical/haematological blood indices or those that had a grand mal seizure with features of preeclampsia and no previous history of a seizure disorder such as epilepsy who were excluded from the study. All women who were above 20 weeks’ gestation and met the above criteria were included in the study. Early neonatal death was recorded as death within 7 days of birth. Maternal death was a death of a pregnant woman who died from complications of severe preeclampsia or eclampsia after 20 weeks’ of gestation, within 42 days post-delivery. The main outcome measures were adverse outcomes. Adverse maternal outcome included maternal mortality or one or more serious complication of major organ morbidity. Adverse perinatal outcome included perinatal death or one or more of 5 min Apgar score < 7, respiratory distress syndrome and admission to neonatal intensive unit.

#### Sample size calculation

Simple proportion formula was used, with the following assumptions 95% Confidence interval (CI) and a margin of error of 5%. In the 3 year period (2016–2018) studied, there were 27,000 deliveries that were analysed. The overall incidence of severe preeclampsia/eclampsia at the unit was found to be 1.3% [10]. The final sample size was 386.

#### Data collection

Data were collected by a paper data collection tool. Data were extracted from hospital case notes, registers in labour ward, neonatal intensive care unit, special care baby unit and from mortality registers. Data collected included the socio-demographic information, therapeutic interventions, maternal and perinatal outcomes.

#### Data analysis

Data were entered into Microsoft Excel Inc., then exported to SPSS Version 20 for analysis. Univariate statistics were performed and presented as frequencies and percentages for categorical variables. Mean and standard deviation (SD) were reported for normal data. Bivariate correlations of association between variables were performed using Pearson Chi-square test. A *p* value of < 0.05 was considered statistically significant. Those variables that had a p < 0.2 from the bivariate analysis were considered for binary logistic regression, with Hosmer–Lemeshow goodness of fit and 95% confidence interval used to identify risk factors associated with adverse maternal and perinatal outcomes. A p < 0.05 was taken as statistically significant.

### Results

#### Socio-demographic characteristics of participants

There were 378 women for analysis, with 9 sets of twins giving a total of 387 babies for neonatal analysis. The mean age was 27.32 years (SD ± 7.36), the mean parity was 1.5 (SD ± 1.5) and the mean gravidity was 2.44 (SD ± 1.49). The mean gestational age was 32.61 weeks (SD ± 4.71) and the mean birthweight was 1832.58 g (SD ± 1302.30). Most of the descriptive statistics can be seen in Tables 1 and 2.

**Table 1:** Socio-demographic characteristics of study patients (N = 378)

Variable	Frequency	Percentage (%)
Marital status		
Single	274	72.5
Married	103	27.2
Divorced	1	0.3
Gestational age/weeks		
20–26^+6^	41	10.8
27–29^+6^	60	15.9
30–33^+6^	136	36.0
34–36^+6^	51	13.5
37–39^+6^	71	18.8
> 40	19	5.0
Booking status		
Booked at Mpilo	65	17.2
Booked/referred	213	56.3
Unbooked	100	26.5
HIV status		
Negative	278	73.4
Positive	37	89.9
Unknown	63	16.7

**Table 2:** Fetal characteristics (N = 387)

Variable	Frequency	Percentage (%)
Fetuses		
Singletons	369	97.6
Twins	9	2.4
Birthweight/g		
0–500	17	4.4
501–1000	72	18.6
1001–1500	89	23.0
1501–2000	51	13.2
2001–2500	77	19.9
> 2500	81	20.9
Outcomes		
Live	316	81.6
FSB	6	1.6
MSB	65	16.8
Apgar scores n = 316		
1 min > 7	86	27.2
1 min < 7	129	40.8
5 min < 7	101	32.0
Neonatal outcomes n = 316		
Early neonatal deaths	72	22.8
Discharged home	244	77.2

#### Determinants of adverse outcomes

Table 3 shows binary logistic regression, performed holding other variables constant and showed the following; mothers who had a baby born at 27–29^+6^ weeks’ of gestation were 8 times more likely to be associated with adverse maternal outcomes compared to mothers who gave birth at 37–39^+6^ weeks’ of gestation (OR 8.187, 95% CI 1.680–39.911, *p *= 0.02).

**Table 3:** Risk factors associated with adverse outcomes

Variable	p-value	Odds ratio	Confidence interval	
			Lower	Upper
Maternal outcomes				
Gestational age 37–39^+6^ weeks	Reference			
Gestational age 27–29^+6^ weeks	0.02	8.187	1.680	39.911
Platelet > 150 × 10^9^/l	Reference			
Platelet 0–49 × 10^9^/l	0.001	46.429	17.778	121.253
Platelet 50–99 × 10^9^/l	0.001	18.681	8.873	39.333
Platelet 100–149 × 10^9^/l	0.002	4.147	1.655	10.396
Birth weight 2001–2500 g	Reference			
Birth weight 0–5001 g	0.001	6.339	2.518	15.961
Birth weight 501–1000 g	0.001	5.164	2.099	12.700
Perinatal outcomes				
Maternal complications-absent	Reference			
Maternal complications-present	0.001	3.535	2.087	5.987
Platelet > 150 × 10^9^/l	Reference			
Platelet 0–49 × 10^9^/l	0.001	3.690	1.752	7.775
Platelet 50–99 × 10^9^/l	0.0001	2.952	1.639	5.317
Corticosteroids-not given	Reference			
Corticosteroids-given	0.05	0.479	0.223	1.026
Birth weight 2001–2500 g	Reference			
Birth weight 0–500 g	0.04	74.443	3.987	1390.00
Birth weight 501–1000 g	0.001	157.442	31.132	796.226
Birth weight 1001–1500 g	0.001	27.445	6.901	109.139
Birth weight 1501–2000 g	0.011	6.812	1.563	29.683

Mothers with platelet counts of 0–49 × 10^9^/l were 46 times more likely to be associated with adverse maternal outcomes compared to mothers with normal counts of more than 150 × 10^9^/l (OR 46.429, 95% CI 17.778–121.253, *p *= 0.001). Mothers who had platelet counts of 50–99 × 10^9^/l counts were 19 times more likely to be associated with adverse maternal outcomes compared to those with platelet counts above 150 × 10^9^/l (OR 18.681, 95% CI 8.873–39.33, *p *= 0.001) and those with platelet counts of 100–149 × 10^9^/l were 4 times more likely to be associated with adverse maternal outcomes compared to those with platelet counts above 150 × 10^9^/l (OR 4.147, 95% CI 1.655–10.396, *p *= 0.002).

Mothers who gave birth to babies with birth weights of 0–500 g were 6 times more likely to be associated with adverse maternal outcomes compared to mothers who gave birth to babies with birth weights of 2001–2500 g (OR 6.339, 95% CI 2.518–15.961, *p *= 0.001), and those mothers whose babies weighed 501–1000 g were 5 times more likely to be associated with adverse maternal outcomes compared to those whose babies weighed 2001–2500 g (OR 5.164, 95% CI 2.099–12.700, *p *= 0.001).

Those mothers who had complications were 4 times more likely to be associated with adverse maternal outcomes compared to those mothers had no complications (OR 3.535, 95% CI 2.087–5.987, *p *= 0.001).

Platelet counts were also determinants of adverse perinatal outcomes. Mothers with platelet counts of 0–49 × 10^9^/l were 4 times more likely to be associated with adverse perinatal outcomes compared to mothers with platelet counts of above 150 × 10^9^/l (OR 3.690, 95% CI 1.752–7.775, *p *= 0.001). Mothers with platelet counts of 50–99 × 10^9^/l were 3 times more likely to be associated with adverse perinatal outcomes compared to those mothers with platelet counts of above 150 × 10^9^/l (OR 2.952, 95% CI 1.639–5.317, *p *= 0.0001).

Babies with birth weights of 0–500 g were 74 times more likely to be associated adverse perinatal outcomes compared to babies born weighing more than 2000 g (OR 74.443, 95% CI 3.987–1390.006, *p *= 0.04). Babies with birth weights of 501–1000 g were 157 times more likely to be associated adverse perinatal outcomes compared to babies weighing more than 2000 g (OR 157.442, 95% CI 31.132–796.226, *p *= 001). Babies with birth weights of 1001–1500 g were 27 times more likely to be associated adverse perinatal outcomes compared to babies weighing more than 2000 g (OR 27.445, 95% CI 6.901–109.139, *p *= 0.001).

Corticosteroids reduced adverse perinatal outcomes by 52.1% (OR 0.479, 95% CI 0.223–1.026, *p *= 0.05) in those babies whose mothers received them compared to those whose mothers did not.

### Discussion

In this study, mothers who had a baby at 27–29^+6^ weeks’ of gestation were 8 times more likely to be associated with an adverse maternal outcome compared to mothers who had babies born at 37–39^+6^ weeks’ gestation (OR 8.187, 95% CI 1.680–39.911, *p *= 0.02). A study in South Africa of similar settings, found early gestation at admission to be the mostly strongly associated with perinatal death [11]. A study in China, found gestational week at admission and delivery significantly associated with adverse perinatal outcomes [12].

Pregnant women giving birth to babies with birth weights of 0–500 g were 6 times more likely to be associated with an adverse maternal outcome compared to those giving birth to babies weighing more than 2000 g (OR 6.339, 95% CI 2.518–15.961, *p *= 0.001). In this study, the smaller the birth weight the higher the risks of adverse maternal outcomes.

Determinants of adverse perinatal outcome included the presence of maternal complications. Those mothers with complications were 4 times more likely to be associated with adverse perinatal outcomes compared to those mothers who had no complications (OR 3.535, 95% CI 2.087–5.987, *p *= 0.001). This information could be useful to clinicians in their efforts to reduce poor outcomes associated with hypertensive disorders in pregnancy by acting promptly.

Severe thrombocytopenic counts of 0–49 × 10^9^/l were 46 times more likely to be associated with adverse maternal outcomes compared to those with normal counts of more than 150 × 10^9^/l (OR 46.429, 95% CI 17.778–121.253, *p *= 0.001). As platelet counts improve the risks get lower. According to this study, any woman with a low platelet count has higher odds associated with poor maternal outcomes.

Platelet counts were also determinants of adverse perinatal outcomes. Platelet counts of 0–49 × 10^9^/l were 4 times more likely to be associated with adverse perinatal outcomes compared to those with platelet counts of above 150 × 10^9^/l (OR 3.690, 95% CI 1.752–7.775, *p *= 0.001). Thrombocytopenia of less than 100 × 10^9^/l confer higher oddsof poor perinatal outcomes and patients with severe preeclampsia and eclampsia need close monitoring of their platelet counts and this could be used a crude marker of adverse perinatal outcomes.

Babies born weighing 501–1000 g birth were 157 times more likely to be associated with adverse perinatal outcomes compared to those babies born with more than 2000 g (OR 157.442, 95% CI 31.132–796.226, *p *= 001). In this study, it appears that in women with severe preeclampsia and eclampsia, achieving at least a birth weight of 2000 g is very critical in terms of odds of adverse perinatal outcomes.

Corticosteroids were a determinant of adverse perinatal outcome, reducing adverse perinatal outcomes by 52.1% (OR 0.479, 95% CI 0.223–1.026, *p *= 0.05) in those babies whose mothers received them. This is below the two-thirds reduction reported by others [13].

### Conclusion

Determinants of adverse maternal outcomes were; gestational age 27–29^+6^ weeks, platelets counts of 0–49 × 10^9^/l, 50–99 × 10^9^/l, 99–149 × 10^9^/l, birth weight 0–500 g and birth weight 501–1000 g. The determinants of adverse perinatal outcomes were; the presence of maternal complications, platelet counts of 0–49 × 10^9^/l, 50–99 × 10^9^/l, birth weight of 0–500 g, 501–1000 g, 1001–1500 g and 15,001–2000 g. Receiving corticosteroids was also a determinant of adverse perinatal outcomes. These determinants could be used as potential markers for adverse maternal and perinatal outcomes.

## Limitations

The main limitation of this study is that it is a single centre study whose findings may not be generalized to other units whose management of patients with severe preeclampsia and eclampsia may not be the same.

## Data Availability

All data generated and analysed during this study are included in this published article.

## Abbreviations

APH: antepartum haemorrhage
BP: blood pressure
CLASP: Collaborative Low-dose Aspirin Study in Pregnancy
CI: confidence interval
DBP: diastolic blood pressure
DIC: disseminated intravascular coagulation
ENND: early neonatal death
FSB: fresh still birth
FFP: fresh frozen plasma
HAART: highly active antiretroviral therapy
HELLP: haemolysis elevated liver enzymes low platelets
HIV: human immunodeficiency virus
LSCS: lower segment caesarean section
MSB: macerated still birth
OR: odds ratio
PPH: postpartum haemorrhage
SBP: systolic blood pressure
